# SCORPIO: a utility for defining and classifying mutation constellations of virus genomes

**DOI:** 10.1093/bioinformatics/btad575

**Published:** 2023-09-15

**Authors:** Rachel Colquhoun, Ben Jackson, Áine O’Toole, Andrew Rambaut

**Affiliations:** Institute of Ecology and Evolution, School of Biological Sciences, University of Edinburgh, Charlotte Auerbach Road, Edinburgh EH9 3FL, United Kingdom; Institute of Ecology and Evolution, School of Biological Sciences, University of Edinburgh, Charlotte Auerbach Road, Edinburgh EH9 3FL, United Kingdom; Institute of Ecology and Evolution, School of Biological Sciences, University of Edinburgh, Charlotte Auerbach Road, Edinburgh EH9 3FL, United Kingdom; Institute of Ecology and Evolution, School of Biological Sciences, University of Edinburgh, Charlotte Auerbach Road, Edinburgh EH9 3FL, United Kingdom

## Abstract

**Summary:**

Scorpio provides a set of command line utilities for classifying, haplotyping, and defining constellations of mutations for an aligned set of genome sequences. It was developed to enable exploration and classification of variants of concern within the SARS-CoV-2 pandemic, but can be applied more generally to other species.

**Availability and implementation:**

Scorpio is an open-source project distributed under the GNU GPL version 3 license. Source code and binaries are available at https://github.com/cov-lineages/scorpio, and binaries are also available from Bioconda. SARS-CoV-2 specific definitions can be installed as a separate dependency from https://github.com/cov-lineages/constellations.

## 1 Introduction

Genomic sequencing has been extensively used throughout the SARS-CoV-2 pandemic to allow researchers and public health bodies to track the virus in real time as it evolves by gaining mutations, insertions, deletions, and by recombining, e.g. (Korber *et al.* 2020, du Plessis *et al.* 2021, Hodcroft *et al.* 2021, O’Toole *et al.* 2021a). As variation accumulates gradually over time, we can infer the evolutionary history of a virus by constructing phylogenetic trees. For SARS-CoV-2, epidemiologically relevant phylogenetic clusters have been assigned PANGO lineages (Rambaut *et al.* 2020a), and those meeting minimum prevalence and persistence criteria have been assigned Nextstrain clades (Aksamentov *et al.* 2021). However individual mutations or sets of mutations frequently arise independently many times across the phylogeny in response to selective pressures. For example, the 501Y mutation in the spike protein arose independently in lineages B.1.1.7, B.1.351, and P.1 (the Alpha, Beta and Gamma variants) (Martin *et al.* 2021). Sets or *constellations* of mutations provide some selective advantage to the virus, such as increased transmissibility (Zhou *et al.* 2021, Bálint *et al.* 2022) or virulence (Twohig *et al.* 2022), or immune evasion (Garcia-Beltran *et al.* 2021, Jian *et al.* 2022). As a result, over the last few years the WHO has designated a number of Variants of Interest (VOIs) and Variants of Concern (VOCs) (Konings *et al.* 2021) with specific mutation profiles and these have been tracked globally to inform decisions by public health organizations.

Following the discovery of the Alpha variant in November 2020, SARS-CoV-2 sequencing began to have rapid, real-world impact on border closures and public health responses around the world. Researchers around the world used Pangolin (O’Toole *et al.* 2021b), a software tool that can assign a lineage to a given SARS-CoV-2 genome, to rapidly place their data in the context of the global diversity of SARS-CoV-2, and in particular to check if their sequences included variants of concern. Pangolin had two approaches for assigning genomes to lineages—a machine learning classifier using a random forest (called pangoLEARN) and a phylogenetic placement method based on UShER (Turakhia *et al.* 2021). The former had the advantage of being extremely fast: 5 CPU minutes to assign 10 000 sequences with one thread, by comparison with 80 CPU minutes for UShER in October 2021 (https://github.com/cov-lineages/pangoLEARN/issues/32) (see Section 3 for recent figures). However it was more prone to false negatives and false positives, particularly where consensus sequences were of lower quality with ambiguous bases or reference calls at important sites, either due to amplicon dropout or poor bioinformatics pipelines. For many purposes, such as measuring lineage frequency or growth, a less than perfect specificity and sensitivity is acceptable. However, for VOCs, in particular, the results were being used to make clinical and public health decisions and it was clear that a more explicit classification was required.

**Figure 1.**
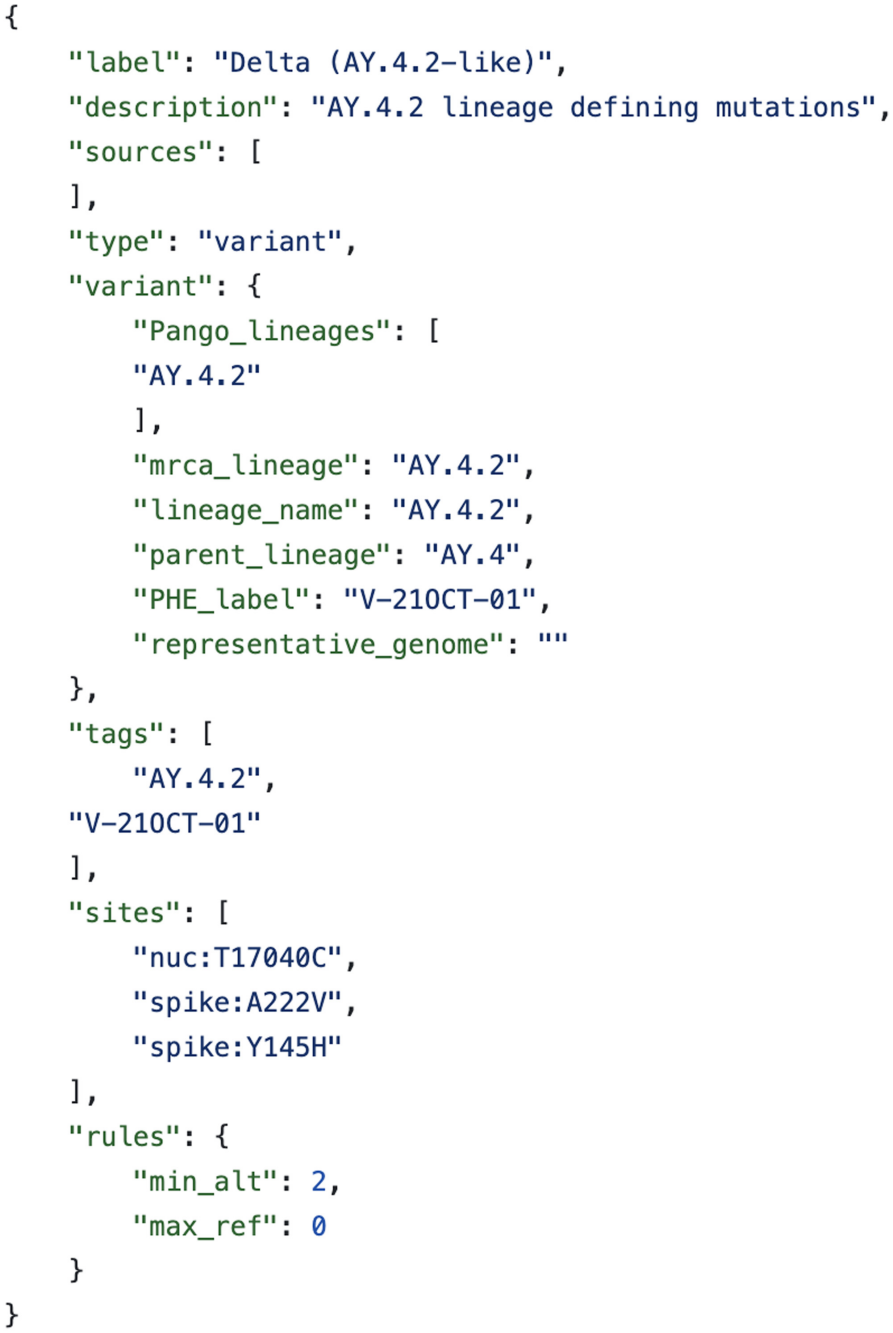
Example constellation definition for variant under monitoring AY.4.2 (Delta). This definition specifies that a sequence must meet the definition for parent lineage AY.4 and in addition have the alternative allele for at least two of the three defining mutations of AY.4.2. This definition does not allow any of the three sites to have a reference allele; an ambiguous base is allowed at up to one site.

Scorpio explicitly types consensus sequences at variant defining sites, allowing for some ambiguity or reference calls, and provides a boolean classification for each constellation of mutations. Scorpio additionally includes tools to aid investigation of sequences which may be misclassified and to define variant constellations.

## 2 The software

The software Scorpio has three main commands: classify, haplotype, and define. The user must provide a reference JSON, detailing the genome sequence of the reference, and providing 1-based coordinates of genes and optionally proteins.

### 2.1 Classify

The primary use of this software is to use explicit mutation typing to classify consensus sequences if they meet the definition of a constellation. Scorpio takes a 1-based reference-coordinate based sequence alignment such as that output by gofasta (Jackson 2022) using “gofasta sam toMultiAlign,” which has become the standard approach for SARS-CoV-2. For each defined constellation it calls alleles at the specified sites, categorizing the call as reference, alternative, ambiguous or another allele. If a genome sequence meets the thresholds specified by the constellation definition, it is classified as belonging to that constellation. The default output is a single summary file, with optional additional columns. Individual counts and True/False classifications for each constellation can be output in individual CSV files. This command is run within pangolin to check the calls made by the pangoLEARN algorithm and to remove both false negative and false positive calls for Variants of Interest and Variants of Concern.

By default, in the event that a sequence meets the criteria for multiple constellations, a winning constellation is chosen by discriminating first based on support (the proportion of defining sites with the alternative allele), then conflict (the proportion of defining sites with the reference allele) and finally based on the number of rules in the constellation definition.

### 2.2 Haplotype

For each sample in the input, Scorpio outputs a barcode for every constellation that represents the concatenation of alleles found at the constellation-defining sites in that sample (in position order). By default, reference alleles are output as a “-” in the string to aid visual scanning for nonreference alleles. This can help to troubleshoot and resolve why a set of samples may fail to be classified as a given constellation, such as amplicon dropout, potential recombination or contamination, or an update required to the constellation definition.

### 2.3 Define

The *define* function allows the user to identify the common mutations within a group of sequences. It takes as input a CSV with a mutation column containing a pipe (“|”) separated list of mutations and indels such as output by “gofasta variants.” If required, the user can specify an outgroup, and those mutations which are common to this outgroup are placed in a separate ancestral site list within the definition, which is then used by classify but not haplotype in order to retain sensitivity whilst removing noise from haplotype barcodes.

### 2.4 Specifying constellations

Constellations are specified by JSON files containing at a minimum: a label (a unique identifying string), sites (a list of defining mutations), and rules (such as a minimum number of alternative calls, a maximum number of reference or ambiguous calls or specific calls required at a given mutation site). Constellations may also specify a parent lineage whose definition must also be met for the constellation to be classified as true (Fig. 1). In practice, these constellation files should be manually reviewed, and rules curated to maximize sensitivity and specificity of classifications based on early consensus sequences.

The general format of a mutation code is: “gene:[ref]coordinates[alt]” where gene is a gene code (or “nuc” for the nucleotide sequence), ref is the nucleotide or amino acids in the reference, alt is the specific nucleotide or amino acids for the alternative allele. Either of ref or alt can be missing if no specific state is required. The intention is for constellation definitions to be human readable, so wherever possible mutation sites are defined relative to the gene or protein, rather than in nucleotides coordinates.

## 3 Context, limitations, and alternatives

An early prototype of Scorpio called “type_variants.py” has been used in pangolin (O’Toole *et al.* 2021b) since December 2020, when the first variants of concern emerged. Scorpio was formally incorporated into Pangolin v3.0 in May 2021 to remove both false negative and false positive calls for VOCs, VOIs and VUMs.

Scorpio provided the essential nonprobabilistic infrastructure which was needed to distinguish variants of concern during the Alpha, Beta, Gamma and Delta waves (Rambaut et al. 2020b, Faria *et al.* 2021, Tegally *et al.* 2021). In December 2021, when Omicron emerged (Viana *et al.* 2022), Scorpio was used to create definitions for Omicron B.1.1.529 and its sub-lineages in a context where many of the early genome consensus sequences had reference bases in regions of low sequencing coverage, or due to contamination with Delta genomes. The first Omicron sequences were initially assigned lineage B.1.1.529, until a second cluster was identified. At this point, the first group were redefined as sublineage BA.1 and the second as BA.2, with the set of shared mutations defining the constellation for putative common ancestor B.1.1.529. Operating within Pangolin, Scorpio classify could identify sequences which matched the definition of B.1.1.529, but not the existing full sets of mutations defining BA.1 and BA.2. Scorpio haplotype was then used to investigate and clarify whether this was due to contamination with Delta, recombination with Delta and to help identify further sublineages of B.1.1.529, later defined as BA.3, BA.4 and BA.5. These classifications allowed the pangolin tool to produce rapid trustworthy classifications when neither pangolin model (pangoLEARN or UShER) was able to.

For most of the SARS-CoV-2 variants, early sequences were heavily scrutinized by groups of researchers and as a result, the sites in the constellation definition can be considered correct by definition. The flexibility comes from the ambiguity allowed by the sets of rules. This allows for lower quality input data, but also naturally gives rise to some false classifications. As new sequences come to light, the definitions should be reviewed and updated to retain accuracy and flexibility.

Since Scorpio was incorporated into pangolin, the primary analysis mode now assigns lineages using placement on a tree with UShER (Turakhia *et al.* 2021) without Scorpio curation post assignment. The careful manual curation of this underlying tree in recent months to handle the many recombinants and convergent mutations has improved accuracy over pangoLEARN for placement of new sequences (de Bernardi Schneider *et al.* 2023), and recent software improvements have made rapid assignments with UShER computationally feasible (it now takes ∼3 CPU minutes to assign 10 000 sequences with pangoLEARN/UShER). Scorpio classification remains a feature within pangoLEARN mode and would be required by any new probabilistic methods.

Over time, Nextclade (Aksamentov *et al.* 2021) has expanded the information it provides to include a full list of mutations present within each sample, although it does not provide a breakdown showing how this compares to defining mutations of variants and it cannot identify defining mutations of a group of sequences.

In its current form, Scorpio assumes that a 1-based reference-coordinate based alignment has been provided, i.e. the alignment is the same length as the reference genome. This alignment by construction does not include insertions with respect to the reference and so no insertions can be typed. Within the context of the SARS-CoV-2 pandemic, the volume of sequence data and the low levels of diversity make this a reasonable simplification and the standard form for multiple sequence alignment. Future development of Scorpio would include extending methods to more general alignments, thereby improving its application to viruses which have been evolving over a longer time, e.g. RSV in response to new treatments and vaccines.

As global restrictions have lifted, we have seen an increase in the number of convergent mutations across the phylogenetic tree, and these are likely to represent adaptation (Ito *et al.* 2023). In the future we hope to apply Scorpio to identifying and classifying specific convergent haplotypes of SARS-CoV-2.

Over the last 2 years, Scorpio and its constellations provided *the* definitions for many of the VOCs and VOIs and the infrastructure which allowed researchers across the world to reliably identify if their samples were a VOC or VOI. Scorpio classifications via Pangolin and GISAID (Elbe and Buckland-Merrett 2017) were used to provide definitive classifications in a nonprobabilistic way. As a result, they ensured that high-level government decisions which had far-reaching consequences—like border closures—were being made on the most accurate assignments possible. In 2021 prior to widespread vaccine rollout, the decisions informed by Scorpio curation delayed introduction of more transmissible variants and delayed peaks in cases, enabling the rollout of vaccines with measures in place to keep COVID-19 cases as low as possible.

## Data avaliability

No new data were generated or analysed in support of this research.
